# A Feasibility Study of an Improved Procedure for Using EEG to Detect Brain Responses to Imagery Instruction in Patients with Disorders of Consciousness

**DOI:** 10.1371/journal.pone.0099289

**Published:** 2014-06-10

**Authors:** Anna Lisa Mangia, Marco Pirini, Laura Simoncini, Angelo Cappello

**Affiliations:** 1 Department of Electrical, Electronic and Information Engineering (DEI), University of Bologna, Cesena, Italy; 2 Rehabilitation Unit, Maggiore Hospital, Bologna, Italy; University of British Columbia, Canada

## Abstract

One of the major concerns of recent studies is the correct discrimination between vegetative and minimally conscious state as the distinction between these two conditions has major implications for subsequent patient rehabilitation. In particular, it would be advantageous to establish communication with these patients. This work describes a procedure using EEG to detect brain responses to imagery instruction in patients with disorders of consciousness. Five healthy subjects and five patients with different disorders of consciousness took part in the study. A support vector machine classifier applied to EEG data was used to distinguish two mental tasks (Imagery Trial) and to detect answers to simple yes or no questions (pre-Communication Trial). The proposed procedure uses feature selection based on a nested-leave-one-out algorithm to reduce the number of electrodes required. We obtained a mean classification accuracy of 82.0% (SD 5.1%) for healthy subjects and 84.6% (SD 9.1%) for patients in the Imagery Trial, and a mean classification accuracy of 80.7% (SD 11.5%) for healthy subjects and 91.7% (SD 7.4%) for patients in the pre-Communication Trial. The subset of electrodes selected was subject and session dependent.

## Introduction

Recent improvements in intensive care have led to an increase in the number of patients who survive severe head trauma, intracranial haemorrhage or non-traumatic anoxic brain injuries. Although some of these patients make a good recovery, the remainder show consciousness disorders on two different levels. In the worst case, patients remain in a persistent vegetative state (VS); others show minimal but definite behavioural evidence of self or environmental awareness, a condition defined as “minimally conscious state” (MCS) [1] [2].

The correct discrimination between VS and MCS has major implications for subsequent care and rehabilitation, but the rate of misdiagnosis has been estimated at between 15% and 40% of cases [3]. Several new techniques, including functional magnetic resonance imaging (fMRI), cognitive event-related potentials (ERPs) and quantitative EEG analysis (QEEG) are currently being developed to assess patients correctly.

Boly and colleagues developed an fMRI paradigm of reliable voluntary activation in response to an imagery task [4]. They found two imagery tasks which elicit extremely reliable, robust and statistically distinguishable patterns of activation in specific regions of the brain: spatial navigation imagery, imagining moving from room to room in your own home, and motor imagery, imagining playing tennis. After this preliminary study, Owen and colleagues used Boly et al.'s paradigms to show that a patient who seemed to be entirely vegetative might be aware, since her blood oxygen-level was modulated by the various mental imagery tasks [5]. With the same technique, Monti and colleagues assessed 54 patients with consciousness disorders using Boly et al.'s imagery tasks as a means of communicating yes or no answers to simple yes or no questions by imaging one task for yes and the other for no [6]. Despite the very promising results obtained by these studies, fMRI-based applications remain challenging for many reasons: 1) high costs; 2) limited scanner availability; 3) the inactive state of these patients; 4) the frequent uncontrolled, involuntary movements inside the scanner; 5) the substantial physical stress to patients on transfer to the fMRI facility. Furthermore, metal implants, including plates and pins, which are common in most traumatically injured populations, rule out the use of fMRI.

By contrast, electroencephalographic (EEG)-based paradigms have many advantages over fMRI for monitoring patients with altered consciousness. Because of its low cost, non-invasiveness and portability, a dedicated bedside system for these patients is feasible. Some previous studies used EEG-based brain computer interface (BCI) systems to validate the scientific reliability of EEG-related procedures in discriminating mental imagery tasks in disabled people [7]. These studies demonstrated that EEG-signals could be produced and controlled by thinking about specific imagery tasks, thereby confirming that this activity could also serve as a new form of communication in patients with disorders of consciousness. With a paradigm similar to that of Monti et al., Cruse and colleagues used EEG to detect command-following in vegetative state patients undertaking two motor imagery tasks (image moving hand and toe). They used EEG-signals recorded by 25 electrodes located over the motor area and a support vector machine (SVM) to classify the two tasks, obtaining a classification accuracy of 61–78% [8]. In a second study, Cruse and colleagues optimized their technique using only four fixed electrodes and confirmed the classification accuracy of their previous study [9]. Cruse et al.'s results were discussed by Goldfine et al. because of the difficulty of the tasks and the critical reliance on certain statistical assumptions. They reanalysed the data with a method independent of such assumptions and reported no evidence for covert consciousness [10]. Using Fisher's linear discriminant approach and two different tasks with respect to those used by Cruse et al. (image swimming and image walking in one's own home), Goldfine et al. investigated whether spatially and spectrally localized changes in EEG power spectra could identify responses to command in healthy subjects and patients with severe brain injury. They demonstrated that imagining the two imagery tasks changed the EEG power spectrum at multiple channels and frequency bands in healthy subjects and patients [11]. We disagree with Goldfine et al.'s consideration on the difficulty of Cruse et al.'s tasks, and think that Goldfine et al.'s tasks are more difficult. For this reason, we used Cruse et al.'s tasks in our study. John and colleagues established the reproducibility of differential EEG source localization during requested imagery tasks in vegetative state patients [12], as had been established for fMRI. Lulé et al. proposed an auditory P3-based BCI to detect command-following in patients with disorders of consciousness [13]. The latest study by De Massari et al. demonstrated the possibility of yes-no communication with an amyotrophic lateral sclerosis (ALS) subject using an EEG signal [14]. This study included 15 healthy control subjects. De Massari et al. developed a semantic classical conditioning paradigm able to discriminate between conditioned yes or no responses in the cortex, and thus enable basic affirmative and negative communication in all subjects. Classification accuracy in the discrimination of answers was 64% in healthy subjects and 62% in the ALS patient. Another study by De Massari et al. found no reliable communication in a completely locked-in state (CLIS) patient, but satisfactory BCI performance in a locked-in state (LIS) patient obtaining an accuracy up to 70% [15]. Kotchoubey and colleagues described a CLIS patient whose slow EEG activity significantly differed between trials when he was asked to try to move the left compared to the right hand [16]. In healthy participants, motor imagery also produces clearly distinguishable modulation of EEG sensorimotor rhythms (SMRs), similar to those seen during motor execution. Kübler and colleagues showed that LIS patients with ALS could learn to modulate their SMRs with *>*70% accuracy, but did not test VS patients with this paradigm [17].

Results obtained in the above studies are often discrepant but confirm the usefulness of EEG to reliably detect awareness in patients with a clinical diagnosis of VS. Therefore, EEG may serve as an important tool for the assessment of awareness components in patients with disorders of consciousness in the clinical setting. In the light of these findings, we set out to improve classification accuracy in yes-no communication with subjects, while reducing the number of electrodes required. We report here the results of a feasibility study conducted on a group of five healthy subjects and five patients with different levels of disorders of consciousness who underwent EEG recording during the execution of two mental imagery tasks. Without any assumption about the positioning of electrodes, the main aim of the study was to see whether some mental activation patterns could be discriminated using only EEG data and simple power parameters extracted from the EEG. A preliminary investigation was conducted on healthy subjects. Results obtained were used to design the experiments with patients. With a view to clinical application, the first step of the study was to simplify the set-up for the acquisition of EEG data by reducing the number of electrodes. The second aim of the study was to evaluate the reliability of a classification procedure to distinguish between the electrode activation patterns of the two mental states evoked by the two imagery tasks. The third was to evaluate the reliability (and hence practical feasibility) of the classification results during communication with the subjects, using the EEG signal to detect answers to simple yes or no questions.

## Methods

### Subjects

Five control subjects and five patients with different levels of consciousness disorders took part in the study. The five control subjects (age 26 to 37) were healthy and free of medication and any central nervous system disorder.

**Figure 1:**
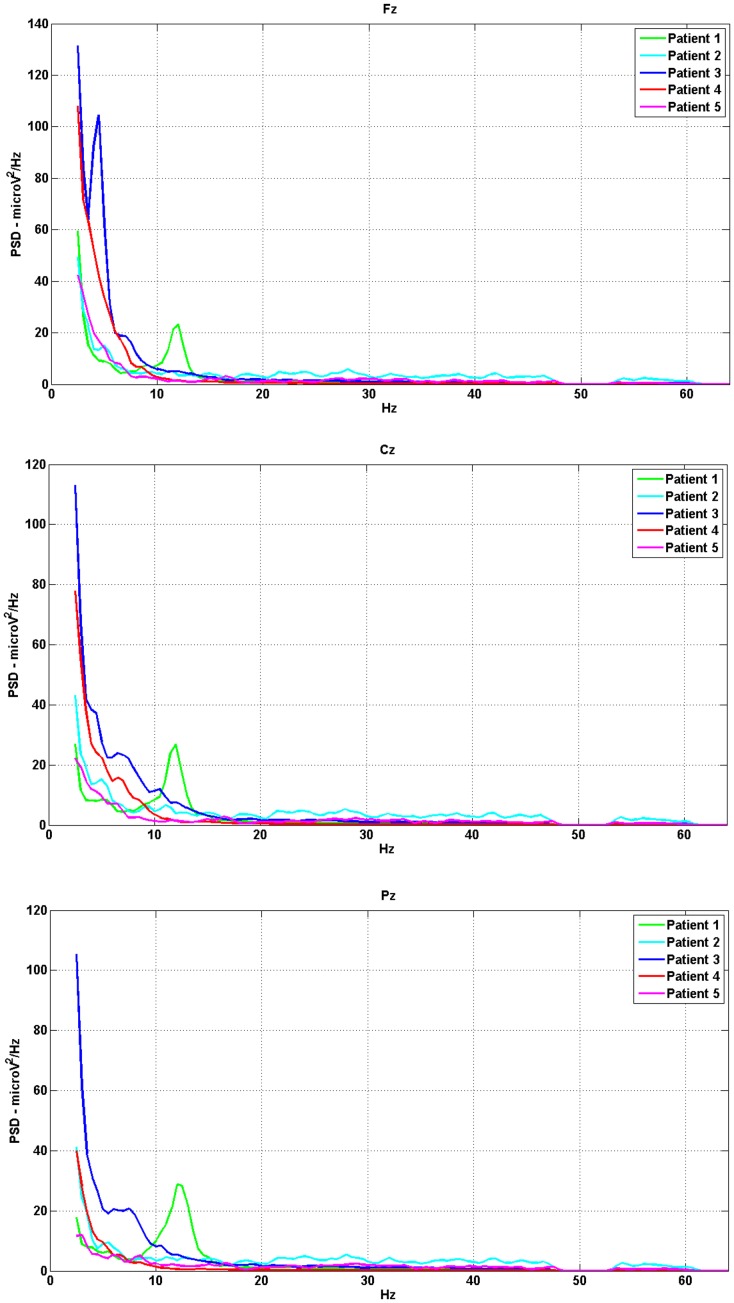
Examples of the EEG PSD in the 5 patients in resting state. The figure shows the PSD spectrum in the frontal (Fz), central (Cz) and parietal (Pz) regions. The alpha peak is visible only in spectra of patient 1, i.e. the only patient in conscious state. All other spectra are characterized by an attenuation of the alpha rhythm. In these spectra there was relatively greater power at lower frequencies and diminished power at higher frequencies. The predominant rhythms are delta and theta, which represent the most prominent abnormality in awake EEGs on VS-MCS patients.

Table 1 lists the demographic and clinical information of the five patients [18]–[19]. In addition, Figure 1 shows the power spectra of patients in resting condition as extracted from 120 s signal recording.

**Table 1:** Demographic and clinical information of the five patients with disorders of consciousness (DoC).

Patient no.	Age at assessment (years)	Sex	Interval post trauma (months)	Aetiology	CRS-R	LCF	DoC classification
**1**	**22**	**F**	**10**	**TBI**	**-**	**7**	**CS**
**2**	**29**	**F**	**22**	**TBI**	**2/1/2/2/1/2**	**3**	**MCS**
**3**	**36**	**M**	**5**	**TBI/Anoxia**	**1/0/2/2/0/2**	**3**	**VS/MCS**
**4**	**63**	**M**	**4**	**TBI**	**2/1/5/1/0/2**	**3**	**MCS**
**5**	**60**	**M**	**17**	**TBI**	**3/4/5/2/1/2**	**4**	**MCS**

CRS-R  =  coma recovery scale-revised; LCF  =  level of cognitive functioning; TBI  =  traumatic brain injury; CS  =  conscious state; MCS  =  minimally conscious state; VS  =  persistent vegetative state.

### Protocol

#### Healthy subjects and imagery tasks

The experiment comprised two sessions repeated on two consecutive days at the same time of day. Each session included two trials: an Imagery Trial and a pre-Communication Trial. The Imagery Trial consisted of ten one minute repetitions of two tasks: one imagining a movement of the right hand and the other imagining a movement of the right foot. The subjects were instructed to mentally simulate the movements (kinaesthetic motor imagery). We chose this internal imagery because several studies demonstrated that good recognition rates are only achieved when the ‘imaginer’ uses the strategy of kinaesthetic motor imagery (first-person process), whereas recognition is almost impossible when the subject forms a visual image of another's action (third-person process) [20]. The sequence of hand and foot imagery was randomized. In the pre-Communication Trial the subjects were asked simple yes or no personal questions (Text S1). Subjects were instructed to imagine for 30 seconds a movement of the right hand for an affirmative answer and a movement of the right foot for a negative answer. The pre-Communication Trial comprised six questions repeated five times. The entire experiment was performed with closed eyes. The subjects' answers were collected after the experiment.

#### Patients

The experiment consisted of one session comprising the same two trials used for the healthy subjects: the Imagery Trial and the pre-Communication Trial. The Imagery Trial consisted of seven 30 second repetitions of the two imagery tasks. In the pre-Communication Trial the patients were asked the same simple yes or no questions used for healthy subjects. The six questions were repeated twice. The patients' answers were collected after the experiment through their relatives. Fewer repetitions were made in patients because of their limited attention span.

The healthy participants and the families of the patients included in the study provided their written informed consent to participate in the study. The Ethical Committee of the Maggiore Hospital and Bologna Health Trust approved the study and consent procedure.

### Recordings and signal preprocessing

EEG was recorded from 31 electrodes (Fp1, Fp2, AF3, AF4, F3, F4, F7, F8, Fc1, Fc2, Fc5, Fc6, Fz, C3, C4, Cp1, Cp2, Cp5, Cp6, Cz, P3, C4, PO3, PO4, Pz, T3, T4, T5, T6, O1, and O2) positioned according to the international 10–20 layout using a Neurowave System (Khymeia, Italy). EEG signals, referenced to linked ear lobes, were sampled at 256 samples/s, and preliminarily band-pass filtered between 3 Hz and 60 Hz. Trial datasets underwent i) manual identification and rejection of artefactual segments, and ii) data cleaning with independent component analysis [21].

For each section, the epochs after the fifth second were eligible for the classification process. Power spectral density (PSD) was extracted from two second epochs without overlap. A modified periodogram method, based on FFT-algorithm and Blackman Harris window, was used. Subsequently, we averaged eight values of the extracted PSD with a six second overlap, thus obtaining one PSD for every 16 seconds. A 16-second epoch length PSD represents a good compromise between reliability and sensitivity with respect to EEG signal variations [22]. The power in four frequency bands was extracted from the calculated PSD value: theta (4–8 Hz), alpha (8–13 Hz), beta (13–25 Hz), and gamma (25–40 Hz). We restricted the analysis to these four bands because these are supposed to be the most active during the performed tasks [23] [24]. *Parameter* was defined as the group of the powers in theta, alpha, beta and gamma bands extracted for each 16-second epoch and each electrode.

For each subject and each session, there are 31 sets of parameters, one for each electrode. Ten parameters were included in each set for the Imagery Trial and five for the pre-Communication Trial. Each value of the variable described above was labelled with the corresponding imagery task.

### Search for the best site

The first aim of the study was to simplify the set-up for the acquisition of EEG data with a view to clinical use of the proposed method. To reduce the number of electrodes, a one-way analysis of variance (ANOVA) on two levels (hand and foot) was performed with a significance level *p*<0.05. ANOVA analysis was carried out for each subject, each session and each electrode-band combination of the Imagery task. The total number of ANOVAs executed was 1240 (5 subjects × 2 sessions × 31 electrodes × 4 bands). We then selected the electrodes showing a significant difference between hand and foot imagination in at least one frequency band for each subject and each session (see Table S1) assigning a unit score to the selected electrodes. Finally, we ordered the electrodes on the basis of the total maximum score of 10 (5 subjects and 2 sessions). Best electrodes (BE) for successive analyses were selected as the eight electrodes with the highest score. After selection of the BE an ANOVA analysis was carried out on two levels (hand and foot) with a significance level *p*<0.05. The ANOVA was performed for each patient and each BE-band combination of the pre-Communication Trial. The total number of ANOVAs executed was 160 (5 patients × 8 electrodes × 4 bands). This analysis aimed to evaluate if the BE selected on the healthy subject also provided a significantly different activation in the two imagery tasks in the patients.

### Classification performance

The second and third aims of the study were 1) to evaluate the possibility of classifying the two mental states corresponding to the two imagery tasks, through an analysis of the Imagery Trial, and 2) to establish a means of communicating with the subject by detecting his/her answer to simple yes or no questions, by analysing the pre-Communication Trial. We thought that the two trials involve different cognitive processes. During the Imagery Trial the subject imagines a definite behaviour, without other contingent activities. During the pre-Communication Trial the subjects are involved in additional mental processes, i.e., initiating and sustaining the will to answer through the imagery activities. Moreover, content-dependent additional emotions and memory-related activations could not be excluded *a priori*. For these reasons we considered the two trials separately.

#### Imagery Trial

A linear SVM classifier (SVMc) was used to find the best hyperplane capable of discriminating between the two classes with the maximum possible margin [25], since this is known to increase the generalization capability [26]. The parameters used for the SVM classifier were a soft margin equal to 1, a linear kernel function and a least-square method to find the separating hyperplane. To obtain an unbiased estimation with small sample sizes, nested leave-one-out cross validation was employed to determine the classifier's generalization error across the entire dataset [27]. The external leave-one-out cross validation (LOO CV) was used to evaluate the final discriminant ability of the classifier through the classification accuracy. This external LOO CV splits the dataset into N different combinations of training and testing sets (TRi, TSi), where N is the size of the dataset. An internal LOO CV was performed on each resulting TRi to find the best subset related to that specific training set. The aim of this work was to select the best group of electrodes to maximize the classification accuracy. For this reason, all possible combinations of these electrodes were considered after preliminary selection of the eight BE. These combinations are all single BE (8), all BE couples (28), all BE triples (56), all the groups of four BE (70), all groups of five BE (56), all groups of six BE (28), all groups of seven BE (8), and all BE considered together. The number of electrodes included in each group was defined as “cardinality” (1, 2, 3, 4, 5, 6, 7, 8).

**Figure 2:**
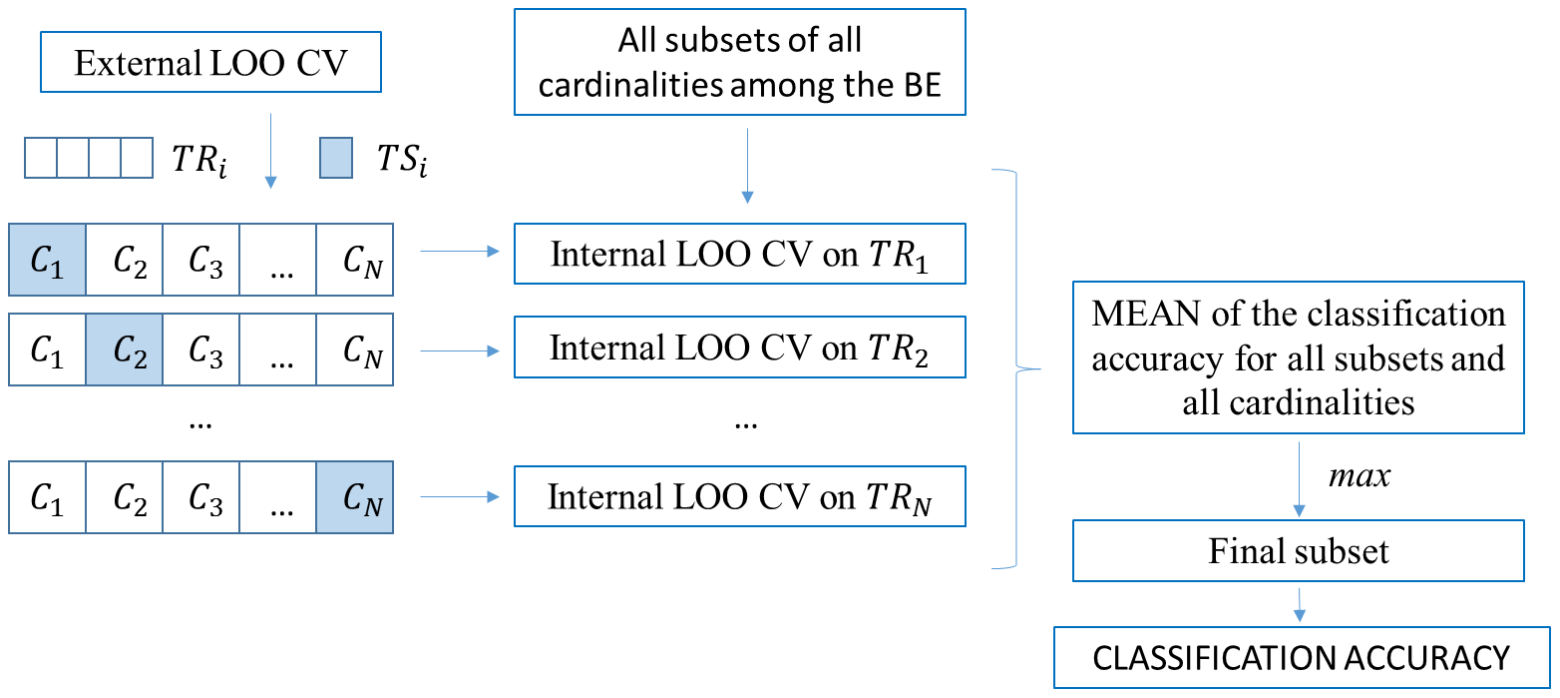
Explanation of the features selection procedure; 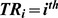 testing set, 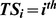 testing set.

An exhaustive search was performed in each internal LOO CV (all subsets of all cardinalities among the BE) to find the subset optimizing the classification performance (see Figure 2). We defined the features of the classification process as the powers of the 32 electrode-band couples (4 bands × 8 electrodes). To make an exhaustive analysis for each subject and patient, and each session of the Imagery Trials, we trained and tested the classifiers using 4-8-12-16-20-24-28-32 features (4 bands × 1-8 electrodes) using all the BE combinations. In each internal loop we trained and tested 8-28-56-70-56-28-8-1 SVMc respectively. The output of the classifiers trained with the Imagery Trial data defined the imagery classification accuracy (ICA). The ICA is the rate of correctly classified parameters (each parameter is the group of the four powers in theta, alpha, beta and gamma bands) in the Imagery Trial using the features selected by the internal loop of the nested LOO. We compared the ICA for both healthy subjects and patients with the random classification level computed with the theoretical method proposed by Müller-Putz et al., with a significance level of 0.05 [28].

#### Pre-Communication Trial

As for the Imagery Trial, we used a linear SVM classifier and a nested cross validation. In this Trial the N different combinations of datasets in which we split the data are the questions and not the single parameters; for the healthy subject N = 30, for the patients N = 12. For each question there are five parameters, so we define the cross validation procedure as Leave-Five-Out Cross Validation (LFO CV). The external LFO CV splits the dataset into N different combinations of training and testing sets (TRi, TSi), where N is the number of questions. An internal LFO CV was performed on each resulting TRi to find the best subset related to that specific training set. An exhaustive search was performed in each internal LFO CV (all subsets of all cardinalities among the BE) to find the subset optimizing the classification performance (see Figure 2). As for the imagery trial, we defined the features of the classification process as the powers of the 32 electrode-band couples (4 bands × 8 electrodes). To make an exhaustive analysis for each subject and patient, we trained and tested the classifiers with 4-8-12-16-20-24-28-32 features (4 bands × 1-8 electrodes) using all the BE combinations. In each internal loop we trained and tested 8-28-56-70-56-28-8-1 SVMc respectively. The external LFO CV was used to evaluate the final discriminant ability of the classifier through the classification error rate.

The output of the classifiers trained with the pre-Communication Trial data defined the communication classification accuracy (CCA). The CCA was computed not in terms of correctly classified parameters, as for the Imagery Trial, but in terms of correctly classified answers. Each answer comprised five parameters, then the class to which answers are attributed is decided by counting the assignment of the parameters in the two classes (yes/no) according to a majority criteria. CCA computation was used to estimate how accurately the classifier will perform with respect to future questions. As for the Imagery Trial, we compared the CCA for both healthy subjects and patients with the random classification level computed with the theoretical method proposed by Müller-Putz et al., with a significance level of 0.05 [28].

### Software tools

MATLAB language and toolboxes were used for data processing and analysis. In particular, we used the Signal Processing Toolbox to preprocess the recorded data, and the Bioinformatics Toolbox for the SVMc classification.

## Results

### Search for the best site

**Figure 3:**
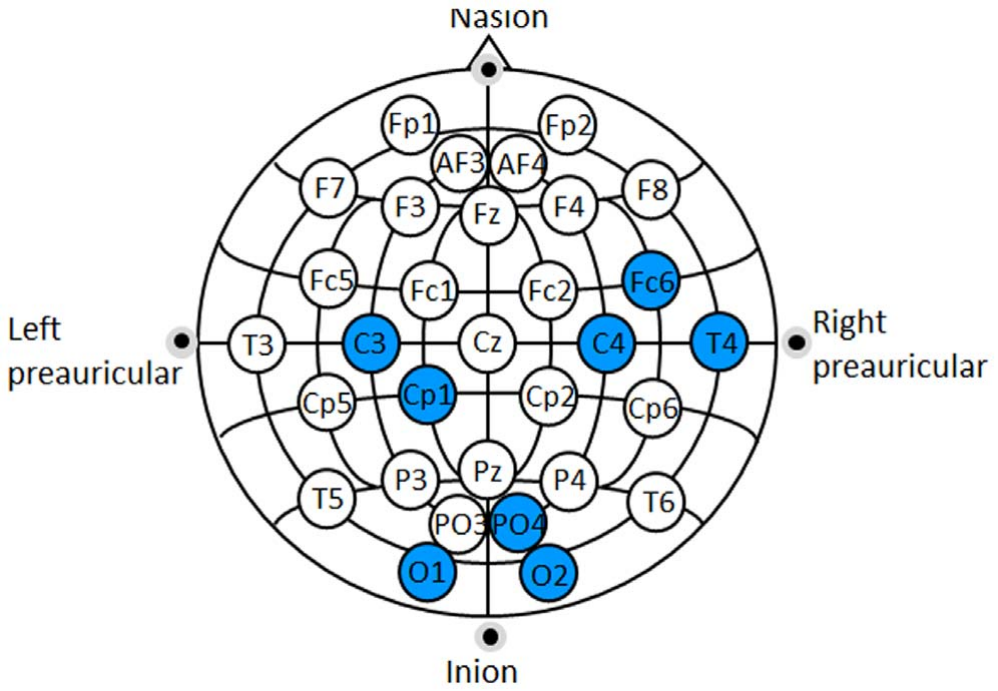
Best electrodes (BE) selected by the ANOVA analysis. All marked electrodes registered a significantly different activation during the two tasks for each healthy subject and each session in at least one frequency band.

Table S1 lists the significant electrodes in at least one band for each subject and each session. As shown in Figure 3, the eight BE selected were: C4, C3, PO4, O2, T4, O1, Fc6 and Cp1. These electrodes will be used in the following analyses. Table S2 shows the couple BE-band with a significantly different activation in the two imagery tasks for the patients in the pre-Communication Trial.

### Classification performance

**Figure 4:**
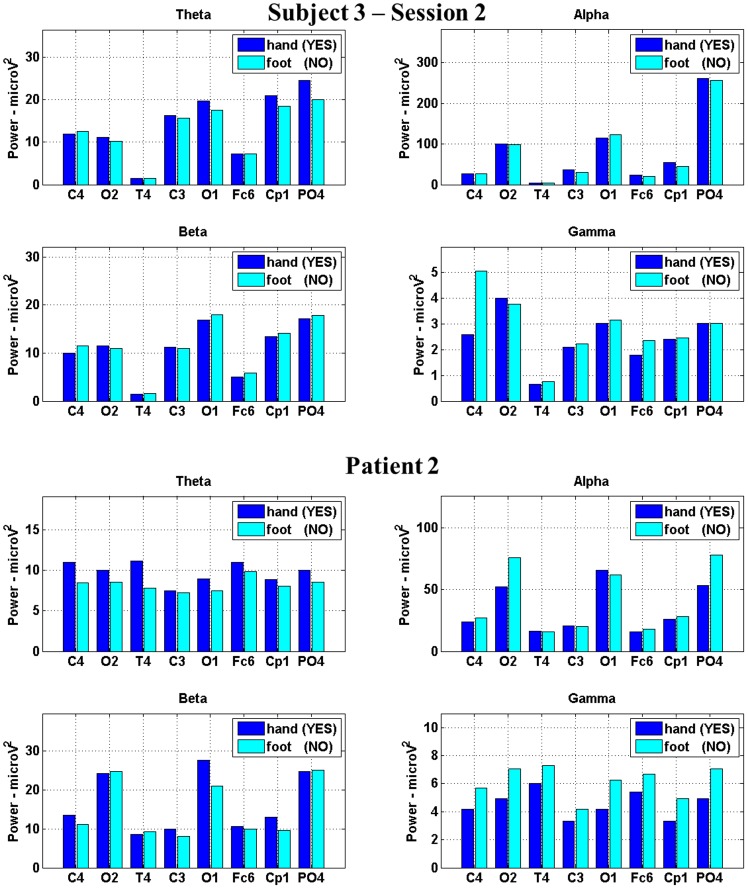
Example of the features used in the classification process for subject 3 in session 2 and patient 2. The features are the powers in theta, alpha, beta and gamma bands extracted by the BE for the two motor imagery tasks.

For each subject and each patient, each session and each trial, we extracted the mean power in theta, alpha, beta and gamma bands for the BE and we trained and tested the classifiers using these features. Figure 4 shows an example of the features used for the classification process for one healthy subject and one patient.

**Figure 5:**
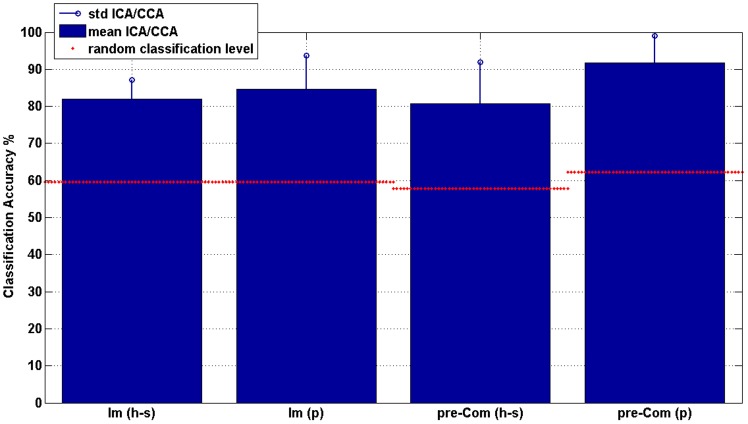
Mean and SD of the classification accuracy of the best configurations for the healthy subjects (h-s) and patients (p) in the Imagery (Im) and pre-Communication (Com) Trials. The classification accuracy was the mean of the accuracy obtained using the best configuration (in terms of electrodes selected by the nested CV procedure) for each subject and each patient. The figure also shows the random classification level computed for healthy subjects and patients and each trial.

Table 2 shows the mean and standard deviation of the best classification accuracy obtained for the healthy subjects and the patients for each cardinality in the Imagery Trial and in the pre-Communication Trial. Tables S3 and S4 show the complete results on the best classification accuracies obtained for each subject, each patient, each session and each cardinality for the Imagery Trial and pre-Communication Trial, respectively. Considering the best configuration for each subject and each patient, we computed the mean ICA and the mean CCA. The mean ICA of the best configurations for each session was 82.0% (SD 5.1%) for healthy subjects and 84.6% (SD 9.1%) for patients. The mean CCA of the best configurations for each session was 80.7% (SD 11.2%) for healthy subjects and 91.7% (SD 7.4%) for patients (see Figure 5). In each case the ICA and CCA were greater than the random classification level with a significant level of 0.05. Table S5 lists the electrode configurations maximising the classification accuracy for each subject, each session and each trial.

**Table 2:** Mean and standard deviation (SD) of the best classification accuracy obtained for the healthy subjects and the patients for each cardinality in the Imagery Trial and in the pre-Communication Trial.

CLASSIFICATION ACCURACY % (Mean±SD)								
Electrodes	one	two	three	four	five	six	seven	eight
**IMAGERY TRIAL**								
**Subjects**	67.2±5.1	74.1±6.0	77.1±6.3	79.2±5.4	**81.7±5.1**	79.8±5.3	79.6±5.0	76.8±5.1
**Patients**	63.4±8.8	76.1±8.4	78.9±9.3	81.9±8.8	83.3±7.8	**83.6±8.5**	**83.6±10.3**	82.4± 9.6
**PRE-COMMUNICATION TRIAL**								
**Subjects**	66.4±17.2	**73.7±14.5**	71.1±15.8	66.4±14	68.1±12.9	66.4±12.1	65.4±16	68.3±8.6
**Patients**	66.7±20.4	**80.1±17.2**	66.7±15.6	65.1±3.7	63.4±9.5	68.4±10.8	70.1±12.6	66.8±11.7

## Discussion

### Search for the best site

#### Neuroanatomical correlation

We did not use a specific algorithm for source localization, so only a qualitative analysis of the detected electrode sites can be made. We found that the BE are mainly located in the centro-parietal and parieto-occipital cortex. This confirms the results of a previous study demonstrating activation of motor cortex, temporo-occipital, parieto-occipital areas and occipital lobe during the execution of motor imagery tasks. Solodkin et al. used fMRI and structural equation modelling to study the activation pattern during motor execution and motor imagery [29]. They demonstrated a predominant activation of the motor and premotor cortex but also an activation of the occipital cortex during the imagery task. Ishizu et al. demonstrated that the act of imaging hand movement activates the extrastriate body area in the lateral occipital cortex [30]. Lebon et al. studied the role of the inferior parietal cortex in the motor circuits, explaining that the inferior parietal lobe is part of an inhibitory network that may prevent unwanted movement during imagery tasks [31]. Szameitat and colleagues investigated the functional neuroanatomical correlates of motor imagery [32]. The participant imagined motor tasks involving the whole body, e.g. swimming: activation was apparent in Brodmann areas 4 and 6, corresponding to the motor cortex. Munzert and colleagues demonstrated activation of the motor area during imagery of dance and gymnastic movements [33]. The pre-motor cortex plays important roles in the planning paradigm, programming and execution of motor acts. Imagined and executed movements often require the same activation to be performed [34], suggesting they are generated through analogous computational steps in the brain. This implies that imagined movements also include a planning/preparation phase before the imagination. The parietal cortex is an important sensory integration hub and its different sub-regions, projecting to various brain areas including the premotor and motor cortex, play important roles during motor execution. In particular the postero-parietal cortex is involved in the visuo-motor transformation process.

#### Clinical relevance

Although EEG has many practical advantages over fMRI, correct positioning of the electrodes is time-consuming and requires skilled personnel. Several studies [35]–[41] investigating selection of the minimum number of channels for classification purposes in BCI systems were able to reduce the number of electrodes required to between 4 and 12. All studies with motor imagery tasks used a pre-fixed set of electrodes positioned over the motor cortex. We did not make any *a priori* assumptions on the positioning of the electrodes, so the electrodes selected were not localized in a single area on the scalp. Nevertheless, the use of fewer electrodes simplifies preparation by unskilled personnel. The preliminary choice of the eight electrodes in healthy subjects, selecting the optimum subject-specific subset also proved suitable for patients and guaranteed a higher classification accuracy of their answers.

### Classification performance

We initially analysed the Imagery Trial finding a mean ICA of 82.0% (SD 5.1%) for healthy subjects and 84.6% (SD 9.1%) for patients considering the BE configuration for each subject and each session. A high ICA in the classification of the two tasks demonstrates that both healthy subjects and patients were able to perform the tasks. Since the two tasks can be reliably differentiated, we think that the patients' (and subjects') level of cognitive activity is sufficient to allow attempts to communicate.

The pre-Communication Trial analysis evaluated the possibility of detecting answers to simple yes or no questions. The search for the best configuration specific for each subject and each session from the BE allowed us to distinguish between the two answers with a mean CCA of 80.7% (SD 11.2%) for healthy subjects and 91.7% (SD 7.4%) for patients.

The search for the optimum subset from the eight BE shows that the best ICA and the best CCA were obtained with different electrode configurations. This variability was found in all subjects and all sessions. In the Imagery Trial, the classification performance for both healthy subjects and patients improved using more than four electrodes, while the use of five electrodes yielded the highest mean accuracy. In the pre-Communication Trial, the classification performance for both healthy subjects and patients improved using two electrodes. The proposed procedure allowed us to fix a robust and statistically significant common subset for all subjects (BE), but we also considered the inter and intra-subject variability by selecting a subject and session specific subset. In a future practical application of our protocol, each communication session will be preceded by a configuration session in which the classification algorithm selects the optimum electrode subset from the fixed BE. Comparing the performance of the healthy subjects with that of the patients we found a higher classification accuracy in the patients. This finding could depend on the simplified brain activity of the patients that allows a simpler representation during the two imagery tasks. The healthy subjects could be thinking about many things besides the imagery task, whereas the patients perhaps could only perform the task by concentrating on it, thus decreasing spurious variability that would lead to decreased ICA and CCA. Lastly, the patients could be more motivated in conducting the experiment.

## Conclusions

This study evaluated the possibility to classify two mental states corresponding to two imagery tasks, using non-event-related EEG techniques, and to use them for communication purposes. Firstly, we outlined a general, automated procedure to identify the BE sites in terms of statistical significance of the PSD features in the two tasks. We paid particular attention to issues related to discrimination between and communication with patients affected by different levels of consciousness disorders. This preliminary study involved just five healthy subjects and five patients and hence did not aim to define a standard protocol for clinical assessment. The proposed automated procedure provided good classification accuracy for the two investigated imagery tasks, while identifying suitable and clearly defined sites for EEG spectral parameters classification. These promising results suggest further studies and investigations, namely: 1) increasing the number of patients who are vegetative or minimally conscious; 2) developing an online procedure to establish communication with the patients.

## Supporting Information

Table S1 Electrodes with significant level of ANOVA (p<0.05) in at least one band for each subject and each session.
(DOCX)

Table S2 The Table shows the results of ANOVA analysis performed for each patient (P) and each BE-band (C4 O2 T4 C3 O1 Fc6 Cp1 PO4 – Theta Alpha Beta Gamma) combination of the pre-Communication Trial (p<0.05). The ANOVA analysis allowed to find the BE-band couples with a significantly different activation during the two imagery tasks, then during the two answers “yes” (y) and “no” (n). The table reports also if the power increases or decreases depending on the answers. It is possible to observe that Theta, Alpha and Gamma bands contribute likewise in the discrimination of the two answers. The Table shows also that “yes” answer, that corresponds to the hand movement imagery, increases the power in the low frequency bands, while “no” answer, that corresponds to the foot movement imagery, increases the power in the high frequency bands.
(DOCX)

Table S3 Best classification accuracy obtained for each subject, each patient, each session and each cardinality for the Imagery Trial.
(DOCX)

Table S4 Best classification accuracy obtained for each subject, each patient, each session and each cardinality for the pre-Communication Trial.
(DOCX)

Table S5 Electrode configurations maximizing classification accuracy for each subject, each patient, each session and each trial.
(DOCX)

Text S1 List of the questions made to the healthy subjects and patients in the pre-Communication Trial.
(DOCX)
