# Supplementary material for: A Feasibility Study of an Improved Procedure for Using EEG to Detect Brain Responses to Imagery Instruction in Patients with Disorders of Consciousness
Source: PLoS One. 2014 Jun 10;9(6):e99289. doi: 10.1371/journal.pone.0099289 (PMC4051659; doi:10.1371/journal.pone.0099289)
Supplement: Table S1 — Electrodes with significant level of ANOVA (p<0.05) in at least one band for each subject and each session. (DOCX) [file pone.0099289.s001.docx]

**Table S1: Electrodes with significant level of ANOVA (p<0.05) in at least one band for each subject and each session.**

|  | **Session 1** | **Session 2** |
| --- | --- | --- |
| **Subject 1** | F4 – C4 – O2 – F8 – T4 – T6 - C3 – O1 – T3 – Cz – Pz – Fc2 – Fc6 – Cp1 - Cp2 - Cp6 – PO4 | F4 – C4 – P4 – O2 – T4 – T6 – Fp1 – F3 – C3 - P3 – O1 – F7- T3 – T5 – Fz – Cz – Pz – AF3 – AF4 – Fc5 – Fc1 – Fc2 – Fc6 – Cp5 – Cp1 – Cp2 – Cp6 – PO3 – PO4 |
| **Subject 2** | Fp2 – C4 – P4 – O2 – F8 – T4 – T6 – Fp1 – C3 – P3 – O1 – F7 – T5 – Fz – Cz – Pz – AF3 – AF4 – Fc1 – Fc2 – Fc6 - Cp5 – Cp1 – Cp2 – Cp6 – PO3 – PO4 | C4 – O2 – T4 – T6 – Fp1 –F3 – C3 – P3 - F7- T3 – Fz – AF3 – Fc5 – Fc1 –Fc6 – Cp6 – PO4 |
| **Subject 3** | Fp2 – F4 – C4 – O2 – F – T4 – Fp1 F3 – C3 – O1 – F7 – T3 – AF3 – AF4 – Fc5 – Fc1 – Fc6 –Cp1 – PO3 – PO4 | Fp2 - C4 – P4 – O2 – F8 – T4 – Fp1 –F3 – C3 – O1 - F7- T3 – T5 – Fz – Cz – Pz – AF3 – AF4 – Fc5 – Fc1 – Fc2 – Fc6 – Cp5 – Cp1 – Cp2 – Cp6 – PO4 |
| **Subject 4** | Fp2 – F4 – C4 - O2 – F8 – T4 – T6 – Fp1 – F3 – C3 - P3 – O1 – F7- T3 – T5 – Fz – Cz – Pz – AF3 – AF4 – Fc5 – Fc1 – Fc2 – Fc6 – Cp5 – Cp1 – PO3 – PO4 | C4 – P4 –O2 – F8 - T4 – T6 – Fp1 - C3 - P3 – O1 – F7 - T3 – T5 - Pz – AF3 – Fc5 – Cp5 - Cp1 – Cp2 – Cp6 – PO3 – PO4 |
| **Subject 5** | Fp2 – C4 – P4 – Fp1 – C3 – P3 – O1 – T3 – T5 – Cz – Pz – AF3 - Cp5 - Cp1 – Cp2 – Cp6 – PO3 – PO4 | Fp2 – F4 – C4 – P4 - O2 – F8 – T4 - C3 - P3 – O1 – T5 – Cz – Pz – AF4 - Fc2 – Fc6 – Cp5 – Cp1 – Cp6 – PO3 – PO4 |
