# Supplementary material for: A Feasibility Study of an Improved Procedure for Using EEG to Detect Brain Responses to Imagery Instruction in Patients with Disorders of Consciousness
Source: PLoS One. 2014 Jun 10;9(6):e99289. doi: 10.1371/journal.pone.0099289 (PMC4051659; doi:10.1371/journal.pone.0099289)
Supplement: Table S2 — The Table shows the results of ANOVA analysis performed for each patient (P) and each BE-band (C4 O2 T4 C3 O1 Fc6 Cp1 PO4 – Theta Alpha Beta Gamma) combination of the pre-Communication Trial (p<0.05). The ANOVA analysis allowed to find the BE-band couples with a significantly different activation during the two imagery tasks, then during the two answers “yes” (y) and “no” (n). The table reports also if the power increases or decreases depending on the answers. It is possible to observe that Theta, Alpha and Gamma bands contribute likewise in the discrimination of the two answers. The Table shows also that “yes” answer, that corresponds to the hand movement imagery, increases the power in the low frequency bands, while “no” answer, that corresponds to the foot movement imagery, increases the power in the high frequency bands. (DOCX) [file pone.0099289.s002.docx]

**Table S2: The Table shows the results of ANOVA analysis performed for each patient (P) and each BE-band (C4 O2 T4 C3 O1 Fc6 Cp1 PO4 – Theta Alpha Beta Gamma) combination of the pre-Communication Trial (p<0.05).** The ANOVA analysis allowed to find the BE-band couples with a significantly different activation during the two imagery tasks, then during the two answers “yes” (y) and “no” (n). The table reports also if the power increases or decreases depending on the answers. It is possible to observe that Theta, Alpha and Gamma bands contribute likewise in the discrimination of the two answers. The Table shows also that “yes” answer, that corresponds to the hand movement imagery, increases the power in the low frequency bands, while “no” answer, that corresponds to the foot movement imagery, increases the power in the high frequency bands.

| **p<0.05(*)** | **C4** | **O2** | **T4** | **C3** | **O1** | **Fc6** | **Cp1** | **PO4** |
| --- | --- | --- | --- | --- | --- | --- | --- | --- |
| **Theta** | P3 (y>n)  P4 (y>n)  P5 (y>n) | P4 (y>n) | P3 (y>n)  P4 (y>n)  P5 (y>n) | P5 (y>n) | P5 (y>n) | P3 (y>n)  P4 (y>n)  P5 (y>n) |  | P3 (y>n)  P4 (y>n) |
| **Alpha** | P1 (n>y)  P4 (y>n) | P2 (n>y)  P4 (y>n) | P1 (n>y)  P3 (n>y)  P4 (y>n) | P4 (y>n) |  | P1 (n>y)  P4 (y>n) | P4 (y>n) | P2 (n>y)  P4 (y>n) |
| **Beta** |  |  | P1 (n>y) | P2 (y>n) | P5 (n>y) |  | P2 (y>n) | P3 (n>y) |
| **Gamma** | P1 (n>y)  P2 (n>y)  P5 (n>y) | P1 (n>y)  P2 (n>y) | P1 (n>y)  P3 (n>y) | P1 (n>y)  P2 (n>y)  P5 (n>y) | P1 (n>y)  P2 (n>y) | P1 (n>y)  P3 (n>y) | P1 (n>y)  P2 (n>y)  P5 (n>y) | P1 (n>y)  P2 (n>y) |
