# Supplementary material for: A Feasibility Study of an Improved Procedure for Using EEG to Detect Brain Responses to Imagery Instruction in Patients with Disorders of Consciousness
Source: PLoS One. 2014 Jun 10;9(6):e99289. doi: 10.1371/journal.pone.0099289 (PMC4051659; doi:10.1371/journal.pone.0099289)
Supplement: Table S3 — Best classification accuracy obtained for each subject, each patient, each session and each cardinality for the Imagery Trial. (DOCX) [file pone.0099289.s003.docx]

**Table S3: Best classification accuracy obtained for each subject, each patient, each session and each cardinality for the Imagery Trial.**

| **IMAGERY TRIAL (classification accuracy %)** | | | | | | | | | |
| --- | --- | --- | --- | --- | --- | --- | --- | --- | --- |
| **Subjects** | **Sessions** | **Electrodes** | | | | | | | |
|  |  | **one** | **two** | **three** | **four** | **five** | **six** | **seven** | **eight** |
| **Subject 1** | Session 1 | 69.2 | 79.2 | 82.5 | 84.2 | **84.9** | 82.5 | 82.5 | 83.4 |
|  | Session 2 | 66.7 | 74.2 | 80 | 79.2 | **82.4** | 80.9 | 79.2 | 75 |
| **Subject 2** | Session 1 | 75.9 | 78.4 | 84.2 | 85 | **85.8** | 83.4 | 83.4 | 79.2 |
|  | Session 2 | 66.7 | 75.9 | 76.7 | 81.7 | **84.9** | 83.4 | 81.7 | 73.4 |
| **Subject 3** | Session 1 | 66.7 | 72.5 | 77.5 | **84.2** | 84.1 | 83.4 | 83.4 | 80.9 |
|  | Session 2 | 66.7 | 70.9 | 74.2 | 77.5 | 81.6 | 80 | **82.5** | 78.4 |
| **Subject 4** | Session 1 | 70 | 82.5 | 83.4 | 84.2 | **87.4** | 84.2 | 84.2 | 82.5 |
|  | Session 2 | 71.7 | 78.4 | 79.2 | **81.7** | 79.9 | 79.2 | 76.7 | 75.9 |
| **Subject 5** | Session 1 | 59.2 | 63.4 | 65.9 | 69.2 | **70.6** | 67.5 | 69.2 | 66.7 |
|  | Session 2 | 59.2 | 65.9 | 67.5 | 72.5 | **75.7** | 73.5 | 73.4 | 72.5 |
| **Mean±SD** | | 67.2±5.1 | 74.1±6.0 | 77.1±6.3 | 79.2±5.4 | **81.7±5.1** | 79.8±5.3 | 79.6±5.0 | 76.8±5.1 |
| **Patient 1** | Session 1 | 75 | 84.2 | 84.2 | 85.9 | 89.2 | 89.2 | **90** | **90** |
| **Patient 2** | Session 1 | 70 | 81.7 | 87.5 | **89.2** | 88.4 | **89.2** | 88.4 | **89.2** |
| **Patient 3** | Session 1 | 55.9 | 69.2 | 66.7 | 68.4 | **71.7** | 70.9 | 68.4 | 69.2 |
| **Patient 4** | Session 1 | 60.9 | 65 | 70.9 | 77.5 | **78.4** | **78.4** | 77.5 | 75 |
| **Patient 5** | Session 1 | 55 | 80 | 85 | 88.4 | 88.4 | 90 | **93.4** | 88.4 |
| **Mean±SD** | | 63.4±8.8 | 76.1±8.4 | 78.9±9.3 | 81.9±8.8 | 83.3±7.8 | **83.6±8.5** | **83.6±10.3** | 82.4± 9.6 |
