# Supplementary material for: A Feasibility Study of an Improved Procedure for Using EEG to Detect Brain Responses to Imagery Instruction in Patients with Disorders of Consciousness
Source: PLoS One. 2014 Jun 10;9(6):e99289. doi: 10.1371/journal.pone.0099289 (PMC4051659; doi:10.1371/journal.pone.0099289)
Supplement: Table S4 — Best classification accuracy obtained for each subject, each patient, each session and each cardinality for the pre-Communication Trial. (DOCX) [file pone.0099289.s004.docx]

**Table S4: Best classification accuracy obtained for each subject, each patient, each session and each cardinality for the pre-Communication Trial.**

| **PRE-COMMUNICATION TRIAL (classification accuracy %)** | | | | | | | | | |
| --- | --- | --- | --- | --- | --- | --- | --- | --- | --- |
| **Subjects** | **Sessions** | **Electrodes** | | | | | | | |
|  |  | **one** | **two** | **three** | **four** | **five** | **six** | **seven** | **eight** |
| **Subject 1** | Session 1 | 83.4 | **97.7** | 93.4 | 86.7 | 83.4 | 76.7 | 73.4 | 66.7 |
|  | Session 2 | **93.4** | 83.4 | 86.7 | 86.7 | 86.7 | 83.4 | 83.4 | 76.7 |
| **Subject 2** | Session 1 | 56.7 | **70** | **70** | 66.7 | 43.3 | 63.4 | 46.7 | 60 |
|  | Session 2 | 56.7 | **80** | 76.7 | 60 | 76.7 | 60 | 43.4 | 60 |
| **Subject 3** | Session 1 | 50 | **76.7** | 73.4 | 70 | 66.7 | 66.7 | 66.7 | 66.7 |
|  | Session 2 | **90** | 76.7 | 73.4 | 70 | 63.4 | 46.7 | 53.4 | 60 |
| **Subject 4** | Session 1 | **60** | 56.7 | **60** | 53.4 | 50 | 50 | **60** | **60** |
|  | Session 2 | 53.4 | 50 | 43.4 | 46.7 | 73.4 | **76.7** | **76.7** | 73.4 |
| **Subject5** | Session 1 | 73.4 | **86.7** | 83.4 | 73.4 | 73.4 | 76.7 | 83.4 | 83.4 |
|  | Session 2 | 46.6 | 60 | 50 | 50 | 50 | 63.4 | **76.7** | **76.7** |
| **Mean±SD** | | 66.4±17.2 | **73.7±14.5** | 71.1±15.8 | 66.4±14 | 68.1±12.9 | 66.4±12.1 | 65.4±16 | 68.3±8.6 |
| **Patient 1** | Session 1 | 66.7 | 66.7 | 66.7 | 66.7 | 75 | 75 | **83.4** | **83.4** |
| **Patient 2** | Session 1 | 50 | 58.4 | 50 | 66.7 | 58.4 | **83.4** | **83.4** | 58.4 |
| **Patient 3** | Session 1 | 50 | **91.7** | **91.7** | 66.7 | 66.7 | 58.4 | 58.4 | 58.4 |
| **Patient 4** | Session 1 | 66.7 | **100** | 66.7 | 66.7 | 66.7 | 58.4 | 58.4 | 58.4 |
| **Patient 5** | Session 1 | **100** | 83.4 | 58.4 | 58.4 | 50 | 66.7 | 66.7 | 75 |
| **Mean±SD** | | 66.7±20.4 | **80.1±17.2** | 66.7±15.6 | 65.1±3.7 | 63.4±9.5 | 68.4±10.8 | 70.1±12.6 | 66.8±11.7 |
