# Supplementary material for: A Feasibility Study of an Improved Procedure for Using EEG to Detect Brain Responses to Imagery Instruction in Patients with Disorders of Consciousness
Source: PLoS One. 2014 Jun 10;9(6):e99289. doi: 10.1371/journal.pone.0099289 (PMC4051659; doi:10.1371/journal.pone.0099289)
Supplement: Table S5 — Electrode configurations maximizing classification accuracy for each subject, each patient, each session and each trial. (DOCX) [file pone.0099289.s005.docx]

**Table S5*:* Electrode configurations maximizing classification accuracy for each subject, each patient, each session and each trial.**

| **Subject** | **Session** | **Electrodes** | |
| --- | --- | --- | --- |
|  |  | **IMAGERY TRIAL** | **PRE-COMMUNICATION TRIAL** |
| **Subject 1** | Session 1 | O2, T4, O1, Fc6, PO4 | T4, O1 |
|  | Session 2 | C4, T4, C3, O1, Fc6, Cp1 | O1 |
| **Subject 2** | Session 1 | C4, O2, C3, Cp1, PO4 | C4, O1  C4, O1, Fc6 |
|  | Session 2 | C4, O2, C3, O1, PO4 | C4, Fc6 |
| **Subject 3** | Session 1 | O2, T4, O1, Fc6 | O2, PO4 |
|  | Session 2 | C4, O2, T4, C3, Fc6, Cp1, PO4 | C4 |
| **Subject 4** | Session 1 | C4, T4, O1, Fc6, Cp1 | O1  C4, O1, PO4  C4, O2, T4, C3, O1, Cp1, PO4  C4, O2, T4, C3, O1, Fc6, Cp1, PO4 |
|  | Session 2 | C4, O2, O1, Fc6 | C4, O2, C3, Fc6, Cp1, PO4  C4, O2, T4, C3, Fc6, Cp1, PO4 |
| **Subject 5** | Session 1 | C4, T4, O1, Fc6, PO4 | C4, Cp1 |
|  | Session 2 | C4, O2, C3, O1, Fc6 | C4, O2, T4, C3, Fc6, Cp1, PO4  C4, O2, T4, C3, O1, Fc6, Cp1, PO4 |
| **Patient 1** | Session 1 | C4, T4, C3, O1, Fc6, Cp1, PO4  C4, O2, T4, C3, O1, Fc6, Cp1, PO4 | C4, O2, T4, O1, Fc6, Cp1, PO4  C4, O2, T4, C3, O1, Fc6, Cp1, PO4 |
| **Patient 2** | Session 1 | O2, T4, Fc6, Cp1  C4, O2, T4, C3, Cp1, PO4  C4, O2, T4, C3, O1, Fc6, Cp1, PO4 | C4, O2, T4, C3, O1, Fc6  C4, O2, T4, C3, O1, Fc6, PO4 |
| **Patient 3** | Session 1 | T4, O1, Fc6, Cp1, PO4 | O1, Cp1  C3, O1, Cp1 |
| **Patient 4** | Session 1 | T4, O1, Fc6, Cp1, PO4  O2, T4, O1, Fc6, Cp1, PO4 | O2, PO4 |
| **Patient 5** | Session 1 | C4, O2, T4, C3, Fc6, Cp1, PO4 | C4 |
