# Supplementary material for: A Feasibility Study of an Improved Procedure for Using EEG to Detect Brain Responses to Imagery Instruction in Patients with Disorders of Consciousness
Source: PLoS One. 2014 Jun 10;9(6):e99289. doi: 10.1371/journal.pone.0099289 (PMC4051659; doi:10.1371/journal.pone.0099289)
Supplement: Text S1 — List of the questions made to the healthy subjects and patients in the pre-Communication Trial. (DOCX) [file pone.0099289.s006.docx]

**Text S1: List of the questions made to the healthy subjects and patients in the pre-Communication Trial**.

1. Do you have more than 30 years?
2. Are you a women?
3. Are you born in Bologna?
4. Are you marriage?
5. Do you have brown eyes?
6. Are you taller than 1.60 meters?

The questions were made in the original language of the subjects and the patients, the Italian language.

The six question were repeated five times for the healthy subjects and twice for the patients.
